# Exposure of *Cryptococcus neoformans* to low nitrogen levels enhances virulence

**DOI:** 10.1007/s10123-024-00504-y

**Published:** 2024-03-14

**Authors:** Caylin Bosch, Barbra Toplis, Anton DuPreez Van Staden, Heinrich Volschenk, Carine Smith, Leon Dicks, Alfred Botha

**Affiliations:** 1 Department of Microbiology, University of Stellenbosch, Private Bag X1, Matieland, Stellenbosch, 7602 South Africa; 2 Division of Clinical Pharmacology, Department of Medicine, Faculty of Medicine and Health Sciences, University of Stellenbosch, Stellenbosch, South Africa

**Keywords:** *Cryptococcus neoformans*, Nitrogen limitation, *Galleria mellonella*, Macrophages, Titanisation

## Abstract

Previous studies have shown a correlation between nitrogen levels and *Cryptococcus neoformans* pathogenicity. Here we report on the in vivo effects of cryptococcal pre-exposure to ecologically relevant nitrogen levels. *C. neoformans* H99 was cultured in yeast carbon base (YCB) supplemented with 0.53 g/L NH_4_Cl and 0.21 g/L NH_4_Cl, respectively, and used to infect larvae of the Greater Wax moth, *Galleria mellonella*. Cells cultured in low nitrogen YCB (LN) were more virulent compared to cells cultured in high nitrogen YCB (HN). Microscopic examination of haemolymph collected from infected larvae revealed that cells cultured in LN were larger than cells cultured in HN, with the majority of LN cells exceeding 10 µm and possibly entering titanisation. Additionally, compared to HN-cultured cells, fewer LN-cultured cells were engulfed by macrophages. The enhanced virulence of LN-cultured cells was attributed to the increased cell size in vivo. In contrast, reduced macrophage uptake was attributed to increased capsule thickness of in vitro cells. Not only do these findings demonstrate the effects of culture conditions, specifically nitrogen levels, on *C. neoformans* virulence, but they also highlight the importance of isolate background in the cryptococcal-host interaction.

## Introduction

*Cryptococcus neoformans* causes life-threatening meningoencephalitis in immunocompromised individuals, with mortality rates reaching up to 75% in developing countries (Rajasingham et al. 2017). Although the species is ubiquitous, it is predominantly isolated from trees and guano (Khan et al. 2007; Gutch et al. 2015; Ellabib et al. 2016) where it is exposed to a multitude of environmental and competition-mediated stresses (Casadevall 2008). While many of these stressors are shared among habitats, each ecological niche presents unique challenges to cryptococcal survival. As such, the microevolutionary effects of such adaptation have stimulated research interest in recent years.

Arras et al. (2017) demonstrated that isolate origin is a key determinant of virulence by reporting on increased virulence of *C. neoformans* strain H99 after passage through animal models. Similarly, Yu et al. (2020) reported variations in the expression of virulence genes for cryptococci isolated from different environments. It was suggested that said variation may ultimately provide clues as to why cryptococci originating from certain ecological backgrounds are better adapted to cause disease.

Concluded from a number of studies (Lee et al. 2011; Frazzitta et al. 2013; Vitale et al. 2012; Bosch et al. 2020; Yu et al. 2020), the availability and quantity of nitrogen play a key role in virulence. Moreover, the expression of virulence-associated genes was recently shown to be modulated by nitrogen limited conditions (Bosch et al. 2021). From these studies, it is clear that environmental conditions, especially nitrogen levels, play a key role in cryptococcal pathobiology. However, while many of the in vitro effects of cryptococcal exposure to low nitrogen concentrations have been elucidated, the in vivo effects of adaptation to nitrogen poor conditions are yet to be determined. In this study we examined how pre-exposure to nitrogen limitation affects the virulence of *C. neoformans* in a *Galleria mellonella* (Greater Wax moth) infection model. We also report on cryptococcal-macrophage interactions. The study provides valuable insights into the effect that nitrogen limitation has on the pathobiology of *C. neoformans* and emphasises the role of isolate background.

## Methods and materials

### Yeast strain and maintenance

All experiments were performed using *C. neoformans* H99, which was revived from glycerol stocks and maintained on yeast-malt extract (YM) agar (Yarrow 1998) at 26 °C. The strain was cultured in 5 mL YM for 18 h at 30 °C, whereafter cells were harvested and washed in sterile dH_2_O. Washed cells were inoculated at a final concentration of 1 × 10^6^ cells/mL into 100 mL yeast carbon base (YCB; Difco, Becton Dickinson and Company, NJ, USA) contained in a 1-L conical flask. Nitrogen was supplied as ammonium chloride in all experimentation and added to YCB at 0.53 g/L (HN growth medium) and 0.21 g/L NH_4_Cl (LN growth medium), respectively. Nitrogen concentrations were selected as described by Bosch et al. (2020). Cultures were incubated on an orbital shaker (120 rpm; New Brunswick Scientific Co. Inc.) for 48 h at 30 °C to ensure optimal growth.

### *Galleria mellonella* virulence assay

To determine whether pre-adaptation to low nitrogen conditions affects the virulence of *C. neoformans*, *G. mellonella* larvae (obtained from the Forestry and Agricultural Biotechnology Institute at the University of Pretoria, South Africa) were used as a virulence model. The *G. mellonella* model was chosen instead of a murine model because wax moths have a shorter reproduction cycle and provide results in a shorter time. Furthermore, compared to rodent models, larvae of *G. mellonella* are easier to maintain, less expensive than animal models and do not require ethical approval.

*Cryptococcus neoformans* H99 was cultured in HN and LN media as described elsewhere. Cells were harvested, washed twice with phosphate buffered saline (PBS) to remove the growth media, and adjusted to 1 × 10^6^ CFU/mL in PBS. Greater wax moth larvae (0.2–0.5 g) were randomly divided into four groups, 20 larvae per group: HN group, LN group, PBS control and an untreated control group. Using a 10-µL Hamilton syringe (701N, 26G, Sigma-Aldrich), 5 µL of the adjusted cell suspension from each respective growth condition (HN and LN) was injected once into the second-to-last larval proleg. In preliminary experiments, this area provided the best results. Also, previous studies conducted in our group have shown that sterile HN and sterile LN had no effect on the larvae. PBS was injected as a non-infection control, while larvae in the untreated group were not injected. All larvae were placed in Petri dishes within a well-ventilated container and incubated at 37 °C. Larval survival was monitored and recorded daily for up to 10 days. Larvae showing no movement when touched were considered dead. All experiments were conducted in triplicate.

### Fungal burden quantification

To quantify the fungal burden, three larvae from each infected group were sacrificed for haemolymph extraction. A small incision was made at the second-to-last proleg with a sterile scalpel. Approximately 20 µL haemolymph was sampled and diluted by adding 20 µL sterile PBS. A serial dilution of the diluted haemolymph suspension was prepared in PBS, and 50 µL of each dilution was plated onto birdseed agar (Yarrow 1998) supplemented with 100 mg/L biphenyl (Sigma-Aldrich, St. Louis, MO, USA) and 200 mg/L chloramphenicol (Sigma- Aldrich). Plates were incubated at 30 °C for 2–3 days and examined for the presence of dark brown colonies characteristic of pathogenic cryptococci. Results were recorded as the number of colony forming units (CFUs) per µL haemolymph.

### Measurement of total cell body, cell size and capsule size

To examine whether pre-adaptation to low nitrogen conditions alters the *C. neoformans* capsule size in vivo, the relative capsule thickness was determined according to Firacative et al. (2014). A drop of India ink (Windsor and Newton, London, UK) was added to 10 µL extracted haemolymph, and capsules were visualised using an eclipse E400 light microscope (Nikon, Tokyo, Japan) at 400 × magnification. Micrographs were taken using a DFSII camera system with a digital Slight DS-U3 power pack (Nikon). Relative capsule thickness was determined as described by Rathore et al. (2016). ImageJ software (National Institute of Health, Washington DC, USA) was used to measure the diameter of the whole cell body including the capsule (Dwc) and the diameter of the cell body up to the cell wall (Dcb). Capsule thickness relative to that of the entire cell body was defined as a percentage: {[(Dwc − Dcb)/Dwc] × 100}. Cell growth was poor, and 20 random cells were measured for each of three biological repeats.

### Detection of reactive oxygen species (ROS)

Free radicals are highly reactive and unstable, and excessive amounts of ROS are known to cause cell damage and trigger apoptosis (Dbouk et al. 2019; Carlson et al. 2021). Cryptococci were examined for the presence of endogenous ROS using a 2′,7′-dichlorofluorescein diacetate (DCFDA; Sigma-Aldrich) staining procedure according to Nair et al. (2016). Three larvae from HN and LN groups were sacrificed at 5 days post infection, and 20 µL haemolymph was extracted from each larva as described previously. Haemolymph was diluted with 20 µL PBS, and cryptococci were counted with a haemocytometer for subsequent adjustment to 1 × 10^5^ cells/mL in 20 µM DCFDA. Care was taken to count cells and not particles. Cells were incubated in the dark for 1 h at 37 °C. Fluorescence was visualised using a Nikon Eclipse E400 epifluorescence microscope equipped with appropriate filters for excitation and emission at 485 and 538 nm, respectively.

### Macrophage uptake

To investigate whether cryptococcal pre-adaptation to low nitrogen affects cell-macrophage interactions, we assessed macrophage uptake according to Srikanta et al. (2011). The human monocytic cell line THP-1 was cultured in complete RPMI-1640 (Catalog number 11875119, Gibco, Thermo Fisher Scientific, MA, USA) supplemented with 4.5 g/L glucose, 2 g/L NaHCO_3_, 10 mM HEPES, 10% (v/v) heat activated foetal bovine serum (FBS; Gibco), 100 µg/mL penicillin (Sigma-Aldrich), 100 U/mL streptomycin, 1 mM sodium pyruvate and 50 µM ꞵ-mercaptoethanol. Monocytic cells were passaged every 2 days to maintain cell density between 1.5 × 10^5^ and 1 × 10^6^ cells/mL. To differentiate THP-1 cells, cell numbers were adjusted to 3.4 × 10^5^ cells/mL in complete RPMI-1640 supplemented with 200 nM phorbol 12-myristate 13-acetate (PMA; Sigma-Aldrich). Flat bottom polystyrene 96-well microtiter plates (uncoated) were then seeded with 3.4 × 10^4^ cells per well and incubated at 37 °C/5% CO_2_ for 48 h. After 48 h, the cells were washed three times in fresh complete RPMI-1640 to remove unattached cells, and the cells were incubated for an additional 24 h in complete RPMI-1640 containing 200 nM PMA. After 24 h media was aspirated, the cells were washed with fresh complete RPMI-1640 after which 180 µL fresh complete RPMI-1640 was added and the cells were incubated for an additional 24 h. For uptake assays THP-1 cells were used at a passage of 10–12.

*C. neoformans* H99 was cultured in HN and LN growth media for 48 h as elsewhere described and washed twice in sterile PBS. Cell numbers were adjusted to 6 × 10^6^ cells/mL, washed once in Mcilvaine buffer (Sigma-Aldrich; pH 6.0) and labelled with 100 µg/mL Lucifer Yellow (Thermo Fisher Scientific, MA, USA) prepared in the same buffer. Following agitated incubation at 25 °C for 30 min, stained cells were washed, resuspended in PBS and mixed with one-half volume human serum for opsonisation. After agitated incubation at 37 °C for 30 min, the opsonised cells were washed three times in PBS and resuspended in complete RPMI-1640 to obtain 1 × 10^6^ cells/mL.

For uptake assays, host cells were prepared by washing once in complete RPMI-1640, aspirating the medium and then adding 100 µL of the labelled and opsonised yeast suspension to each well. Plates were incubated at 37 °C/5% CO_2_ for 2 h, and the cells were washed with PBS. Macrophage structures were stained with 2 µg/mL DAPI (Sigma-Aldrich) and CellMask Deep Red Actin tracker (Thermo Fisher Scientific) according to the manufacturer’s instructions, whereafter cells were fixed with formalin solution (neutral buffered, 10% v/v; Sigma-Aldrich). Following incubation at 25 °C for 15 min, fixed stained cells were washed twice in PBS and subsequently imaged using a Zeiss LSM 780 ELYRA PS1 confocal microscope equipped with the filters for excitation and emission of 360/460, 475/535 and 620/460 nm to detect DAPI, Lucifer Yellow and CellMask Deep Red, respectively. Internalised cells were differentiated from attached cells by the presence of well-defined phagocytic vacuoles. The average number of internalised cryptococci was recorded as a percentage (number of macrophages containing yeast/100 THP-1 cells).

### Statistical analyses

Data obtained from fungal burden quantification, cell size and capsule measurement data, as well as data obtained from macrophage uptake assays, were analysed by performing Student’s *t* test using Statistica software (version 13.5.0.17; StatSoft, OK, USA). Kaplan–Meier graphs of data obtained from the *G. mellonella* virulence assay were constructed using XLSTAT software (version 2019.3.2; Addinsoft, New York, USA), and statistical significance was determined using log-rank sum analysis. Significance was set at *P* = 0.05 for all statistical tests.

## Results

### *Galleria mellonella* virulence assay

**Fig. 1:**
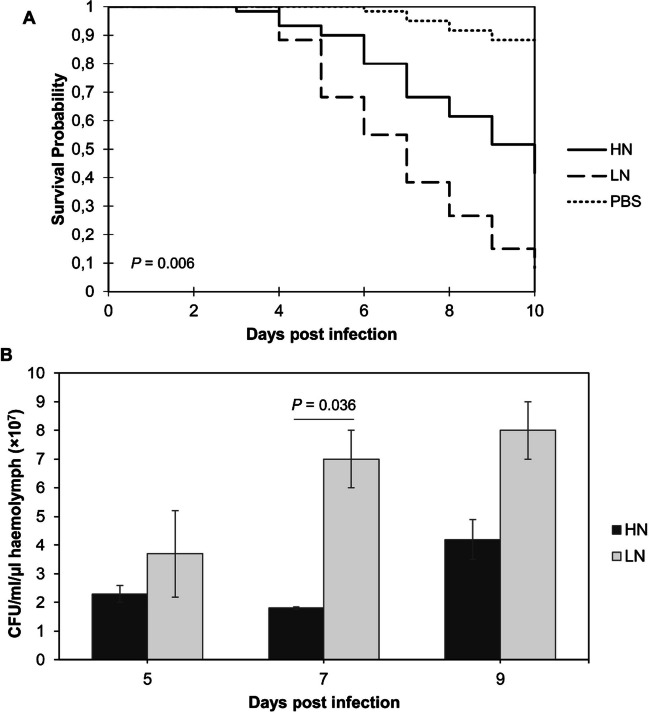
**A** Survival curve of *Galleria mellonella* infected with *C. neoformans* H99. Each curve represents a group of 60 larvae (*n* = 60). Significance level between HN and LN survival as determined by log-rank sum analysis is indicated in the bottom left. **B** Fungal burden of *G. mellonella* larvae infected with *C. neoformans* cells originating from HN and LN growth conditions. Bars represent the mean of three biological repeats. Error bars represent standard error. Indicated significance was determined by a Student’s *t* test

The short life span of *G. mellonella* makes it the ideal model for rapid screenings. More than 1000 articles have been published on PubMed using the *G. mellonella* infection model (Tsai et al. 2016), which demonstrates the increasing popularity of this infection model. Larvae infected with *C. neoformans* H99 showed a steady decline in viability over 10 days, starting 3 to 4 days post infection. Larvae infected with *C. neoformans* H99 pre-cultured in the LN growth medium were significantly (*P* = 0.006) more affected (Fig. 1A). After the 10-day infection period approximately 90% of larvae infected with LN cells were dead and had a dark grey colour, compared to a 60% death recorded for larvae infected with HN cells.

### Fungal burden quantification

Larvae infected with LN cells had a notably higher fungal burden than those infected with HN cells, at 5-, 7- and 9-days post infection (Fig. 1B). An increase in fungal burden was observed among the LN group between day 5 and 7, and a slight increase between day 7 and 9; however, for the HN group, day 7 fungal cell numbers were largely maintained between the first two quantification periods, with an increase only observed between days 7 and 9.

### Determination of in vivo cell size and capsule thickness

**Fig. 2:**
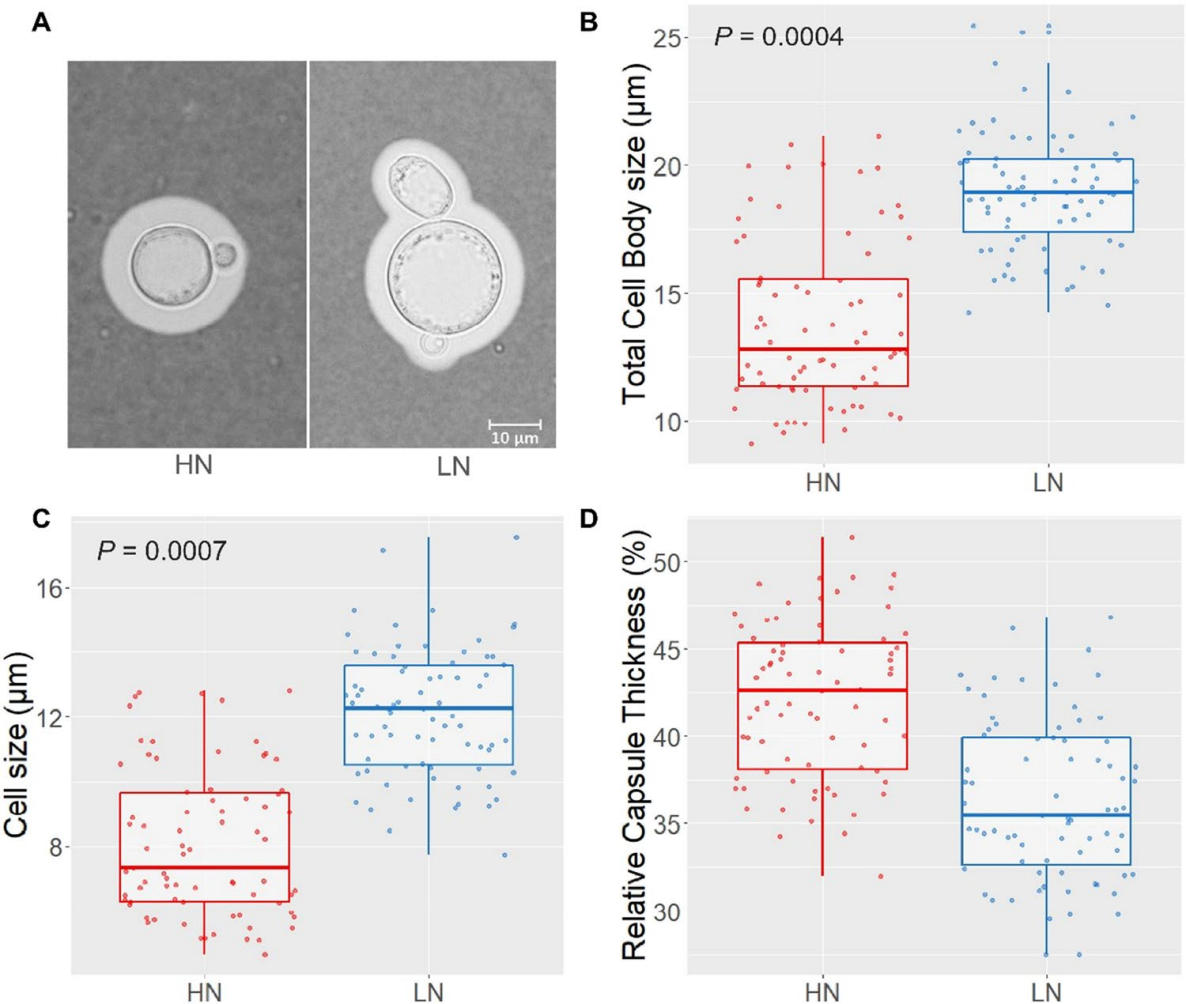
Cell size and capsule thickness of HN and LN-precultured *C. neoformans* cells isolated from infected *G. mellonella* larvae. **A** Micrograph of *C. neoformans* cells in the haemolymph of HN- and LN-infected larvae. **B** Total cell body size (cell size including capsule) of in vivo* C. neoformans* cells. **C** Cell size of *C. neoformans* cells (excluding capsule). **D** Relative capsule thickness of in vivo* C. neoformans* cells (capsule as a percentage of the total cell body). Dots represent 60 random cell measurements from each condition. Indicated statistical significance was determined by Student’s *t* test

The size of *C. neoformans* H99 cells and capsule thickness increased during the infection period. Although the in vivo cell population was heterogenous in size, LN cells were generally larger than HN cells (Fig. 2A), with the size of the total cell body (cell plus capsule) averaging at 18 and 13 µm, respectively (Fig. 2B). Additionally, HN and LN cells differed with respect to cell size (excluding the capsule), where the mean LN cell size was ca. 4 µm larger than HN cells (Fig. 2C). Although HN cells were found to be smaller in size, their capsules were slightly thicker (41% of total cell body size) than LN cells (36% of total body size) (Fig. 2D).

### Detection of reactive oxygen species

**Fig. 3:**
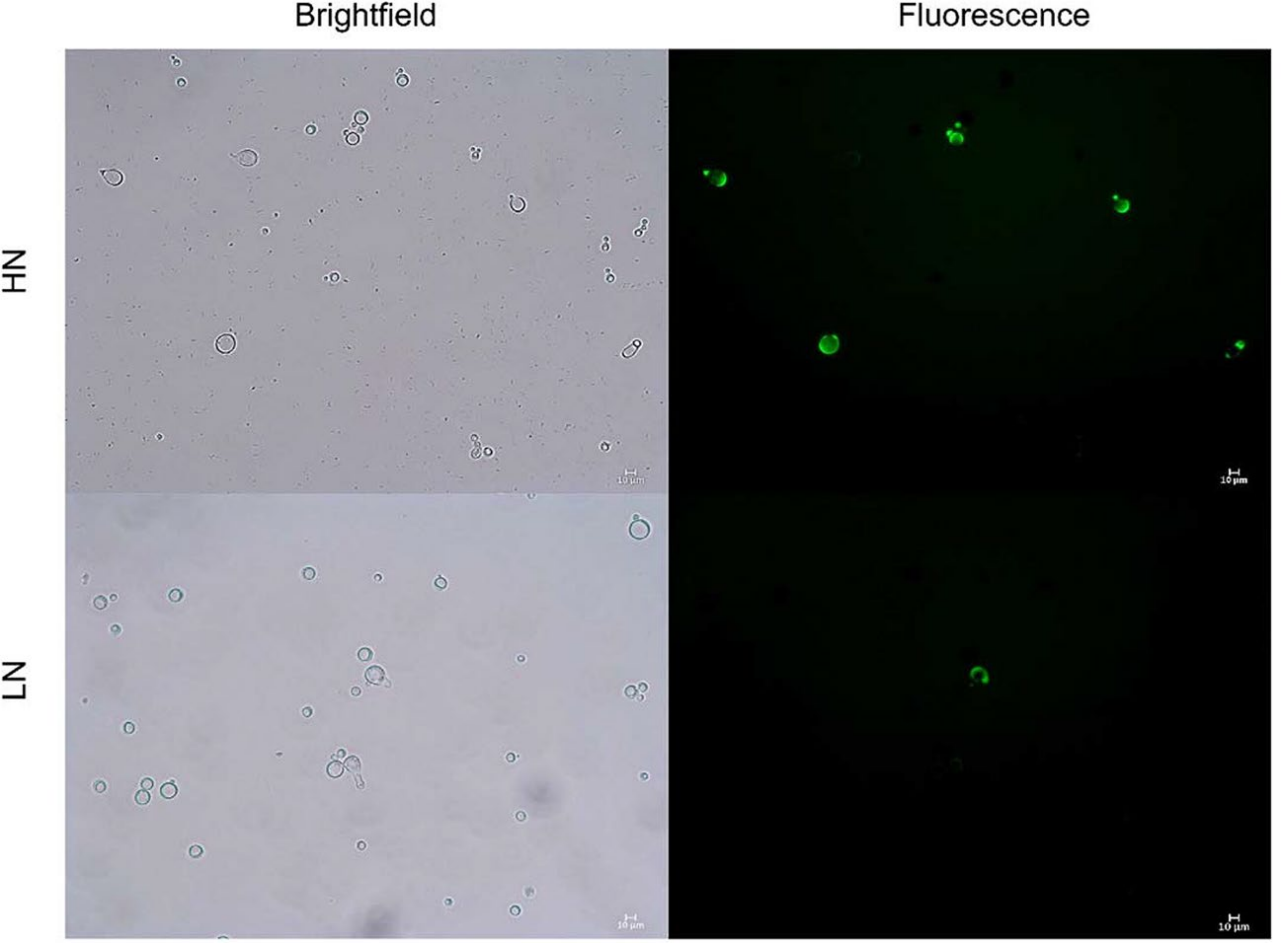
DCFDA stain of *Cryptococcus neoformans* cells isolated from *Galleria mellonella* larvae 5 days post infection. Green fluorescence indicates cells with increased ROS production

Microscopic visualisation of DCFA-stained cryptococci obtained from HN- and LN-infected larval groups revealed that cells originating from HN conditions produced more endogenous ROS in vivo compared to cells originating from LN conditions (Fig. 3).

### Macrophage uptake assay

**Fig. 4:**
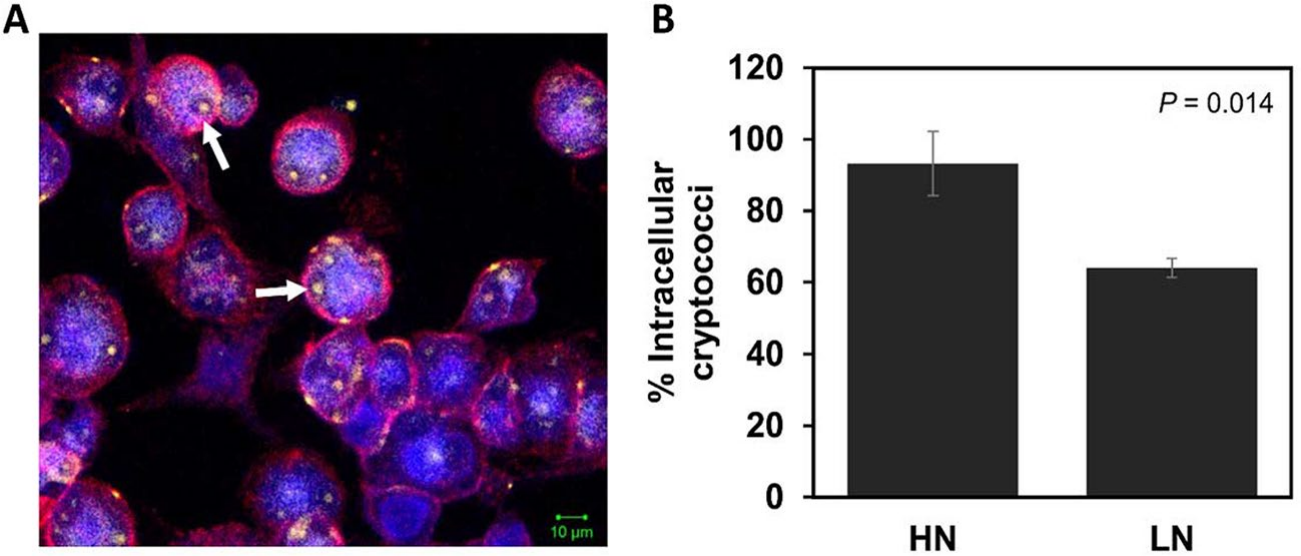
Macrophage uptake assay of HN- and LN pre-cultured *C. neoformans* cells. **A** Fluorescent microscopy image of THP-1 cells stained with CellMask Deep Red and DAPI and incubated with lucifer yellow labelled cryptococci. Internalised cryptococci are indicated by white arrows. Internalised cryptococci were clearly differentiated from attached cells by the presence of well-defined phagocytic vacuoles and were counted separately from those attached to the surface of the macrophages. Magnification = 20 × , Scale bar = 10 µm. **B** Macrophage uptake of HN- and LN-pre-cultured cryptococci. Bars represent the mean of three biological repeats. Error bars represent standard error. Indicated statistical significance was determined by Student’s *t* test

Confocal fluorescent imaging revealed that cryptococcal cells were successfully internalised by macrophages following 2 h of exposure (Fig. 4A), with the halos surrounding phagocytosed yeasts indicating uptake via an endocytosis mechanism. Quantification of intracellular cryptococci showed that the number of internalised cells was significantly higher for HN-precultured cryptococci compared to those pre-cultured in LN conditions (*P* = 0.014), with an average of 93% internalised HN cells compared to 64% internalised LN cells (Fig. 4B).

## Discussion

In the present study, we investigated the in vivo effects of cryptococcal pre-adaptation to low, ecologically relevant nitrogen conditions using a *G. mellonella* infection model. *Cryptococcus neoformans* cells pre-cultured in growth media supplemented with a lower nitrogen concentration (LN cells) were more virulent than those pre-cultured in media supplemented with a higher nitrogen concentration (HN cells). In a recent transcriptomic study from our research group (Bosch et al. 2021), it was revealed that low nitrogen concentrations increase the expression of several cryptococcal virulence-associated genes. The significant upregulation of these genes in LN cells may, therefore, have contributed to the enhanced virulence phenotype. The results reported with the *G. mellonella* infection model need to be repeated using an animal (e.g., rodent) model to determine if there is a difference in lung colonisation between HN and LN cells. It would also be interesting to study the effect of HN and LN cells on the mammalian central nervous system (CNS).

Bosch et al. (2021) reported that genes related to cell wall integrity and oxidative stress tolerance were upregulated in LN conditions. The findings of the present study suggest that both the enhanced cell wall integrity phenotype, as well as the oxidative stress phenotype are maintained during *G. mellonella* infection. Upon India ink staining and microscopic examination of larval haemolymph, we found that the cryptococcal cells pre-cultured in LN conditions were larger, both in total cell size and cell body size, compared to cells originating from HN pre-culture conditions (Fig. 2). Moreover, a larger proportion of LN cells were found to exceed a cell size (excluding capsule) of 10 µm. Pathogenic cryptococci are known for their unique ability to form large (> 10 µm) polyploid cells known as titan cells (Dambuza et al. 2018). These cells have enlarged vacuoles, a thickened cell wall and a tightly compacted capsule. Based on the larger cell size and smaller relative capsule percentage of LN cells compared to HN cells, it is possible that LN pre-culture conditions promote in vivo titanisation more so than HN conditions, supporting previous findings that demonstrate the relevance of pre-growth conditions for titan cell formation (Dambuza et al. 2018).

In a previous study (Bosch et al. 2021), fluorescence-activated cell sorting (FACS) was used to determine the level of polyploidy. In the present study, the low cell numbers of *C. neoformans* did not allow to conduct FACS. Normally high cell numbers are required to set fluorescent thresholds (gating) in the separation of cells. Furthermore, the fast flowing of cells during FACS may shear cells and rupture cell membranes, which may cause blockage and lead to inaccurate sorting of cells. Bosch et al. (2021) previously demonstrated that the increased expression of cell wall integrity genes (e.g. genes encoding chitinases and mannan biosynthetic enzymes), observed in vitro, could possibly favour titanisation in vivo*.* Mukaremera et al. (2018) has shown that titan cells contain more chitin in their inner cell wall, as well as an additional layer of mannan that is not present in regular cryptococcal cells. Although the effect of nitrogen concentration on capsule composition is yet to be elucidated, the reduced relative capsule thickness observed in LN cells in vivo suggests that nitrogen limited conditions may cause alterations to capsule structure and thus requires further investigation.

The formation of these large cryptococcal cells also enables successful evasion of phagocytosis by host immune cells—not just of the titans themselves but of the entire population of cryptococcal cells, thereby playing a critical role in the establishment of infection (Okagaki and Nielsen 2012). The increased fungal burden that we observed in larvae infected with LN cells was, therefore, likely due to the increased cell size which may have contributed substantially to the enhanced virulence phenotype of these cells. Interestingly, during microscopic examination, haemolymph of LN infected larvae appeared to have fewer free-floating haemocytes compared to HN infected larvae (data not shown). This may also have been the result of the larger proportion of titan cells, since titanisation is known to cause alterations in the host immune response (Park et al. 2009; Wiesner et al. 2015).

Intriguingly, DCFDA staining of cryptococci in larval haemolymph revealed greater levels of endogenous ROS in HN cells compared to LN cells (Fig. 3). Recent work by Zhou et al. (2021) found that the accumulation of endogenous ROS is required for the yeast-to-titan transition in *C. neoformans*, but that the detoxification of ROS following the transition plays an equally critical role. The increased proportion of titan-like cells observed for LN cultures could thus be linked to the lower levels of intracellular ROS under these conditions.

Although the findings of the present study are preliminary, they provide valuable insight into factors that regulate cryptococcal pathogenesis, as they demonstrate the importance of isolate origin. The role of nitrogen availability in cryptococcal pathogenesis has been clearly evidenced, as studies have shown that both the nitrogen source and concentration of available nitrogen can regulate cryptococcal virulence factor production (Lee et al. 2011; Bosch et al. 2020). Here we show that these in vitro effects translate to enhanced virulence within a host. *C. neoformans* is known to occur in two predominant ecological niches, namely, pigeon guano and trees, with the quantity of available nitrogen differing substantially between the two habitats (Lee et al. 2011). Though the two different ecologically relevant nitrogen concentrations used in our study did not vary considerably, pre-exposure to either condition still had significant effects on cryptococcal virulence in an invertebrate host, suggesting that even minor differences in pre-infection conditions could have clinical effects. This notion is further supported by results obtained from the macrophage uptake assay, where we found that HN cells were more readily internalised by macrophages compared to LN cells. Macrophages are the first immune cells encountered by cryptococci, and their phagocytic abilities have a dramatic effect on yeast survival in host niches (Bojarczuk et al. 2016). While phagocytosis by macrophages alone is not sufficient for cryptococcal clearance, it is essential for the initial control of cryptococcal infection. Any factors with the potential to influence this initial cryptococcal-macrophage interaction may thus contribute to the establishment of infection.

In our study, the increased uptake of HN cells by macrophages may be attributed to the reduced capsule size compared to that of LN cells (Bosch et al. 2020), since the polysaccharide capsule is known to have antiphagocytic properties (O’Meara and Alspaugh 2012; Vecchiarelli et al. 2013). Alternatively, the possibility that LN conditions promote in vivo titanisation as discussed previously may also explain the reduced macrophage uptake of LN cryptococci, since titan cells are known to resist phagocytosis (Crabtree et al. 2012; Okagaki and Nielsen 2012). Future studies could investigate this hypothesis by evaluating the capacity of nitrogen limited cells to elicit extracellular trap formation in macrophages, since it has been shown that larger pathogens selectively induce this response in immune cells (Branzk et al. 2014; Urban and Nett 2019). It should, however, be noted that cryptococcal-macrophage interactions are extremely complex, and yeast internalisation does not necessarily equate to killing as cryptococci are known to survive within phagocytes for extended periods of time (Tucker and Casadevall 2002; Charlier et al. 2009). Future studies should, therefore, investigate the effects of different pre-culture conditions on the ability of *C. neoformans* to survive and replicate within macrophages over time. Nevertheless, our findings demonstrate the potential of nitrogen availability to impact cryptococcal interactions with host immune cells. Overall, the results presented in the current study highlight the importance of environmental factors in cryptococcal survival and pathogenesis, and reveal the critical role of isolate background in the cryptococcal-host interaction.

An increase in the production of free radicals can lead to the modification of DNA bases (Angele-Martinez et al. 2014), lipid peroxidation and protein carbonylation (Fedorova et al. 2014). A recent study (Dbouk et al. 2019) has shown that an increase in the production of ROS by *C. neoformans* treated with the antifungal drug fluconazole (FLC) induces oxidative stress in *C. neoformans.* Cells with elevated ROS levels were more sensitive to treatment with FLC. The authors have also shown that co-treatment of FLC-induced oxidative-stressed *C. neoformans* with the antioxidant ascorbic acid alleviated growth inhibition (Peng et al. 2018). Antioxidants clearly counteracted the damage caused by FLC-induced ROS. In the case of treatment with FLC, combined with an increase in ROS, the expression of genes in the ergosterol pathway may be repressed (Dbouk et al. 2019), leading to cell death. Ergosterol is an important component of the plasma membrane. We have shown an increase in ROS when *C. neoformans* is cultured in a medium containing high nitrogen levels. This observation may be important in the treatment of *C. neoformans* infections. Future studies on animal models infected with *C. neoformans* and exposed to a combination of different nitrogen conditions, and azole antibiotics, are required to have a better understanding of the role elevated nitrogen levels may have in the treatment of infections.

## Data Availability

The authors confirm that the data supporting the findings of this study are available within the article.
